# DNA Barcoding Reveals Hidden Genetic Diversity and Environmental Correlates of Lineage Differentiation in *Neozavrelia* Goetghebuer & Thienemann, 1941 (Diptera: Chironomidae)

**DOI:** 10.3390/insects17080814

**Published:** 2026-08-05

**Authors:** Tong-Yin Xie, Yan Zhang, Yi-Zhu Chen, Zheng Liu, Xiao-Long Lin

**Affiliations:** 1 College of Plant Protection, Northeast Agricultural University, Harbin 150030, China; xietongyin@163.com; 2 Shanghai Universities Key Laboratory of Marine Animal Taxonomy and Evolution, College of Fisheries and Life Science, Shanghai Ocean University, Shanghai 201306, China; zhang080100@gmail.com (Y.Z.); cyz42912@gmail.com (Y.-Z.C.); 3 Laboratory of Geo-Specimens Study and Testing, Geological Museum of China, Beijing 100034, China; 4 Department of Stratigraphy and Paleontology, Geological Museum of China, Beijing 100034, China

**Keywords:** *COI* DNA barcodes, species delimitation, ecological niche

## Abstract

Species delimitation and biodiversity assessment in Chironomidae are frequently hindered by morphological conservatism and widespread cryptic diversity. *Neozavrelia* (Diptera: Chironomidae), a genus inhabiting specialized freshwater microhabitats such as springs and hygropetric seepages, remains poorly understood with respect to its genetic diversity and the environmental processes underlying lineage differentiation. Clarifying lineage diversity is a prerequisite for evaluating the ecological factors associated with genetic differentiation. In this study, we compiled a global *COI* DNA barcode dataset comprising 137 sequences from newly generated specimens and publicly available records in the Barcode of Life Data Systems (BOLD). Genetic diversity was evaluated using neighbor-joining (NJ) analysis and Automatic Barcode Gap Discovery (ABGD), whereas environmental and geographic drivers of genetic differentiation were assessed using Mantel tests, distance-based redundancy analysis (db-RDA), variation partitioning, and hierarchical partitioning. A total of 26 molecular operational taxonomic units (MOTUs) were identified, with most lineages forming well-supported clades in the NJ tree. Genetic differentiation was significantly associated with both environmental and geographic distances, although environmental variables explained a substantially greater proportion of the observed variation. Hierarchical partitioning identified slope, annual cloud cover, mean precipitation amount of the coldest quarter, and frost frequency as the principal environmental predictors of lineage differentiation. Our findings reveal substantial *COI* genetic diversity within *Neozavrelia* and suggest that environmental heterogeneity, particularly variation in water availability and thermal conditions, is significantly associated with lineage differentiation. This study expands the global *COI* DNA barcode reference library for *Neozavrelia* and provides new insights into ecological and evolutionary factors associated with diversification in freshwater chironomids.

## Simple Summary

*Neozavrelia* is a genus of small freshwater non-biting midges whose species are often difficult to distinguish because of their highly conserved morphology. This study employed DNA barcoding to investigate the genetic diversity of *Neozavrelia* across Asia, Europe, and North America. Molecular analyses identified 26 distinct genetic lineages, revealing previously unrecognized genetic diversity within the genus and suggesting the presence of multiple putative cryptic lineages. We further evaluated the relative effects of environmental and geographic factors on genetic differentiation. Environmental variables, particularly slope, annual cloud cover, cold-season precipitation, and frost frequency, explained more variation in genetic differentiation than geographic distance alone. These findings highlight that local environmental conditions are strongly associated with lineage genetic differentiation and provide a valuable DNA barcode reference for future taxonomic, ecological, and biodiversity studies of freshwater chironomids.

## 1. Introduction

Freshwater ecosystems harbor exceptionally high biodiversity and provide essential ecosystem functions, including energy flow, nutrient cycling, and organic matter processing. Among freshwater organisms, aquatic insects play key roles in ecosystem functioning and are widely used in biological monitoring because of their sensitivity to environmental change [1,2,3,4]. Chironomids (Diptera: Chironomidae) are particularly valuable bioindicators owing to their high abundance, ecological diversity, and broad distribution [5,6]. However, species identification based solely on morphology is often challenging because of their small body size, complex life cycles, and extensive cryptic diversity [4,7,8,9,10,11,12,13]. Consequently, DNA barcoding based on the mitochondrial cytochrome *c* oxidase subunit I (*COI*) gene has become an effective tool for species identification, cryptic species discovery, life-stage association, and biodiversity assessment in Chironomidae [13,14,15,16,17].

**Figure 1:**
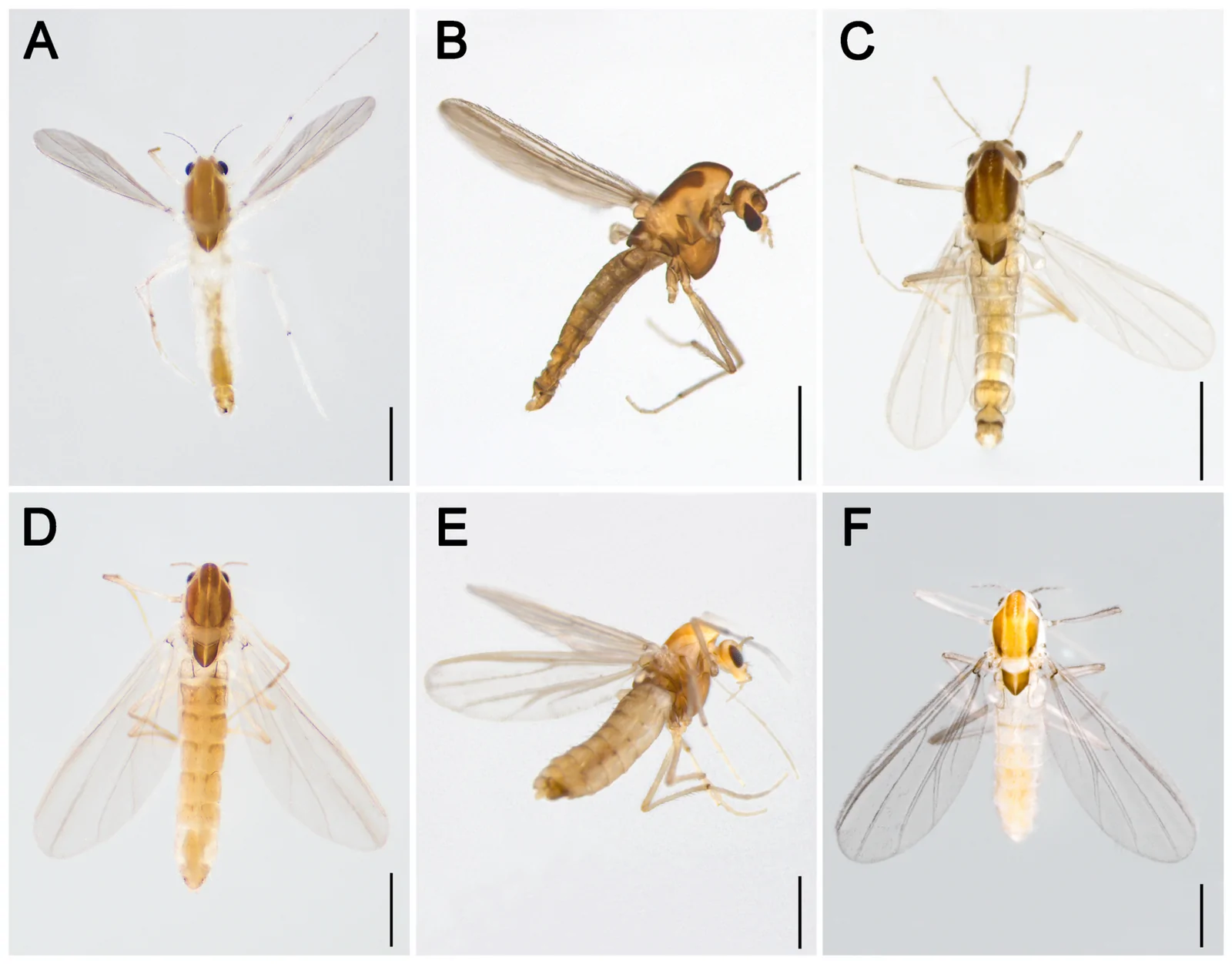
Photos of *Neozavrelia*: (**A**) adult male of *Neozavrelia lindbergi* Reiss, 1968; (**B**) adult male of *Neozavrelia pilosa* Guo & Wang, 2005; (**C**) adult male of *Neozavrelia* sp. 9; (**D**) adult female of *Neozavrelia lindbergi*; (**E**) adult female of *Neozavrelia oligomera* Wang & Zheng, 1990; (**F**) adult female of *Neozavrelia* sp. 2. Scale bars = 0.5 mm. Photos were taken by Xiao-Long Lin, Shanghai Ocean University.

*Neozavrelia* Goetghebuer & Thienemann, 1941 is a small but taxonomically challenging genus within the tribe Tanytarsini [18,19] (Figure 1). The genus is distributed across most major zoogeographic regions except the Afrotropical and Neotropical realms, with East Asia representing an important center of diversity [18,20,21,22,23,24,25]. Recent taxonomic studies have documented considerable morphological variation and revealed several previously unrecognized species, suggesting that the diversity of *Neozavrelia* remains incompletely understood and may include substantial cryptic diversity [20,24,26].

Species of *Neozavrelia* are typically associated with specialized freshwater habitats, including streams, springs, rivers, and hygropetric seepages [20,22,24,27]. Larvae and pupae frequently inhabit permanently wet rock surfaces and seepage zones [24,26]. Such ecological specialization suggests that environmental conditions may strongly influence dispersal, population connectivity, and lineage diversification. Recent observations further indicate that the ecological niche breadth of the genus may be wider than previously recognized, encompassing a variety of warm and humid freshwater microhabitats [28]. These habitat associations provide an excellent opportunity to investigate how environmental variation contributes to genetic differentiation in freshwater insects [24,29].

Specimen identification in *Neozavrelia* has traditionally relied on morphological characteristics of adult males, including antennal flagellomere number, venation and setation patterns of wings, and fine structures of male genitalia such as the anal point, superior volsella, digitus, median volsella, and inferior volsella [18,20,21]. A systematic taxonomic revision of Chinese *Neozavrelia* redefined the generic diagnosis and described multiple new species [25,30]. Subsequent studies from East Asia and the Russian Far East further expanded species records and clarified taxonomic boundaries, revealing substantial morphological variation among populations [23,24,26].

Despite extensive morphological research, molecular resources for *Neozavrelia* remain scarce [24,31,32]. The first partial *COI* sequences of this genus were generated during the morphological redescription of larvae, pupae, and adults of *N. cuneipennis*, providing an initial foundation for molecular identification [20]. To date, publicly available molecular data remain limited, hindering comprehensive assessments of genetic diversity, lineage structure, and diversification processes within the genus [24].

In particular, large-scale studies integrating molecular and environmental data are still lacking [33,34,35]. Accumulated evidence from freshwater insects demonstrates that climatic conditions, hydrological regimes, and geographical isolation play important roles in shaping genetic structure and diversification patterns [36]. Environmental factors such as temperature seasonality, precipitation variability, groundwater dynamics, and topographic heterogeneity can restrict dispersal and promote local adaptation, ultimately driving lineage divergence [37,38,39,40]. However, the relative strength of associations between geographic isolation, environmental heterogeneity and genetic differentiation remains unclear in *Neozavrelia*, a genus specialized for freshwater seepage habitats [24]. Identifying factors associated with lineage diversification in this group will improve our understanding of biodiversity formation, microhabitat adaptation, and biogeographic pattern development in chironomids [34,40].

In this study, we compiled a comprehensive *COI* barcode dataset for *Neozavrelia* by integrating newly generated sequences with publicly available records from BOLD. Specifically, we aimed to: (1) characterize genetic diversity and lineage structure within *Neozavrelia* using *COI* barcodes; (2) evaluate the relative effects of geographic distance and environmental heterogeneity on genetic differentiation; and (3) identify key environmental variables associated with lineage divergence. By linking molecular variation with environmental and geographic factors, this study provides new insights into the processes shaping diversification in *Neozavrelia* and contributes to the development of DNA barcode resources for Chironomidae.

## 2. Materials and Methods

### 2.1. Specimen Collection and Morphological Identification

A total of 117 *Neozavrelia* specimens were collected from freshwater habitats across China and Italy between 2010 and 2025. Sampling was conducted using Malaise traps, light traps, D-nets, and sweep nets. Adult specimens were preserved in 85% ethanol, and larvae were stored in 95% ethanol. All samples were maintained under dark conditions at a constant temperature of 4 °C for long-term preservation to ensure the integrity of morphological structures and the stability of genomic DNA quality. Detailed sampling information is provided in Table S1.

Preliminary morphological identification was conducted under a stereomicroscope, and diagnostic characters were confirmed under a compound microscope, focusing on mouthparts and male genital structures. Specimen identification followed published taxonomic descriptions and identification keys for *Neozavrelia* [20,21,24,25,26]. Voucher specimens are deposited in the College of Fisheries and Life Science, Shanghai Ocean University, Shanghai, China.

### 2.2. DNA Extraction, PCR Amplification, and Sequencing

Genomic DNA was extracted from muscle tissues of specimens. For adults, thoracic or tarsal muscle tissues were used, whereas for larvae, thoracic and abdominal muscle tissues were collected after removing the digestive tract to avoid contamination. Genomic DNA extraction was performed using the Universal Genomic DNA Kit (CWBIO, Taizhou, China) in strict accordance with the manufacturer’s protocol.

The standard mitochondrial cytochrome *c* oxidase subunit I (*COI*) barcode region was amplified using the universal primer pair LCO1490 and HCO2198 [13,41]. The detailed PCR reaction system and thermal cycling procedures are listed in Table 1 and Table 2. Amplified products were purified and sequenced by Genewiz (Suzhou, China).

All successfully sequenced specimens were subsequently mounted in Euparal following standard chironomid slide preparation procedures to preserve diagnostic morphological structures for further examination [42].

### 2.3. Sequence Processing and Molecular Delimitation

All publicly available *COI* barcode sequences of *Neozavrelia* were retrieved and downloaded from the Barcode of Life Data System (BOLD; http://www.boldsystems.org) on 19 May 2026 [43]. Only sequences longer than 500 bp and free of stop codons or quality flags were retained. After filtering, 20 publicly available sequences were combined with newly generated sequences of *COI* barcodes. The final integrated dataset was named “*COI* barcodes of *Neozavrelia* until 2026 (DS-26NEOZ)” and has been deposited in the BOLD database (DOI: dx.doi.org/10.5883/DS-26NEOZ).

Raw sequencing reads were assembled and quality-controlled using Geneious Prime v2024.0.5 (Biomatters, Auckland, New Zealand) [44]. Multiple sequence alignment was conducted in MEGA 12 (Tamura, Philadelphia, USA) [45] using the built-in MUSCLE algorithm (Edmonds, WA, USA) [46]. To ensure dataset accuracy and reliability, all sequences were translated into amino acids to verify the absence of stop codons or pseudogenes.

Pairwise genetic distances were calculated in MEGA 12 based on the Kimura 2-parameter (K2P) model [47] to generate a genetic distance matrix for subsequent genetic differentiation analysis. The complete deletion method was applied to handle missing sites to minimize biases in genetic distance estimation. A neighbor-joining (NJ) tree [48] constructed based on K2P distances with 1000 bootstrap replicates, using pairwise deletion for missing data. The phylogeny was visualized in FigTree v1.4.4 (Rambaut, Edinburgh, UK).

Automatic Barcode Gap Discovery (ABGD) (Marseille, France) [49] was employed for molecular species delimitation based on the NJ phylogenetic framework. The K2P genetic distance matrix was used as the input dataset, and the minimum prior intraspecific divergence (*P*_min_) was set to 0.005 (analysis date: 23 May 2026) to identify potential molecular operational taxonomic units (MOTUs).

### 2.4. Extracting and Reducing the Dimensionality of Geographic and Environmental Data

Geographic coordinate data of all sampling sites were compiled and analyzed in R v4.3.2 (Vienna, Austria) [50]. Spatial distribution patterns were visualized using the packages ape [51], sf [52], and phytools [53].

Environmental variables included 19 bioclimatic variables, frost frequency and duration, aridity index, cloud cover, groundwater depth, elevation, and slope [54,55,56]. All environmental data were obtained from publicly accessible databases, including CHELSA (https://www.chelsa-climate.org/), CGIAR-CSI (https://csidotinfo.wordpress.com/), and EarthEnv (https://www.earthenv.org/) databases.

To reduce multicollinearity, variables were first screened using Pearson correlation analysis (|r| > 0.7), followed by variance inflation factor (VIF) analysis (VIF ≤ 10). Retained variables were standardized using Z-score transformation.

Principal component analysis (PCA) was performed using the prcomp function in R v4.3.2 (Vienna, Austria) to summarize environmental gradients. The first two principal components (PC1 and PC2) were used to represent major environmental variation among sampling sites.

### 2.5. Statistical Analyses of Genetic Differentiation

Relationships among genetic differentiation, environmental variation, and geographic distance were assessed using Mantel and partial Mantel tests implemented in the vegan package in R v4.3.2 (Vienna, Austria) [57]. Genetic distances were based on K2P matrices. Environmental distances were calculated using Euclidean distances of standardized variables, and geographic distances were computed using the Haversine formula [58]. Significance was evaluated using 9999 permutations.

Distance-based redundancy analysis (db-RDA) was used to evaluate the influence of environmental variables on genetic differentiation [59]. The K2P distance matrix was used as the response variable, environmental variables as explanatory variables, and geographic coordinates as conditioning variables to control spatial autocorrelation [60]. Model significance was tested using permutation procedures.

Variation partitioning analysis was conducted to quantify the independent and joint explanatory contributions of environmental and geographic factors [61]. Hierarchical partitioning analysis implemented via the rdacca.hp package was further used to evaluate the relative importance of individual environmental variables based on adjusted R^2^ values [62]. All analyses were performed in R v4.3.2.

## 3. Results

### 3.1. Dataset Characteristics and Geographic Distribution

A total of 137 *COI* barcode sequences of *Neozavrelia* were obtained in this study, with sequence lengths ranging from 524 bp to 658 bp. Among these, 25 sequences represented the full-length 658 bp barcode fragment. Quality control screening confirmed the absence of stop codons or frame-shift mutations in all sequences.

Sampling sites spanned latitudes from 0.91° S to 51.99° N and longitudes from 75.78° W to 137.47° E, covering Asia, Europe, and North America. Geographically, domestic sampling covered multiple biogeographic regions across China, including South China (Hainan, Guangdong, Zhejiang), Southwest China (Yunnan, Guizhou, Sichuan), the southeastern margin of the Qinghai–Tibet Plateau (Motuo and Linzhi, Xizang Autonomous Region), and Northeast China (Heilongjiang, Jilin, Liaoning). Extracontinental specimens were collected from Indonesia (Sumatra), Japan, Greece, the Netherlands, Germany, Sweden, Italy, and the United States.

### 3.2. Species Delimitation via ABGD

**Figure 2:**
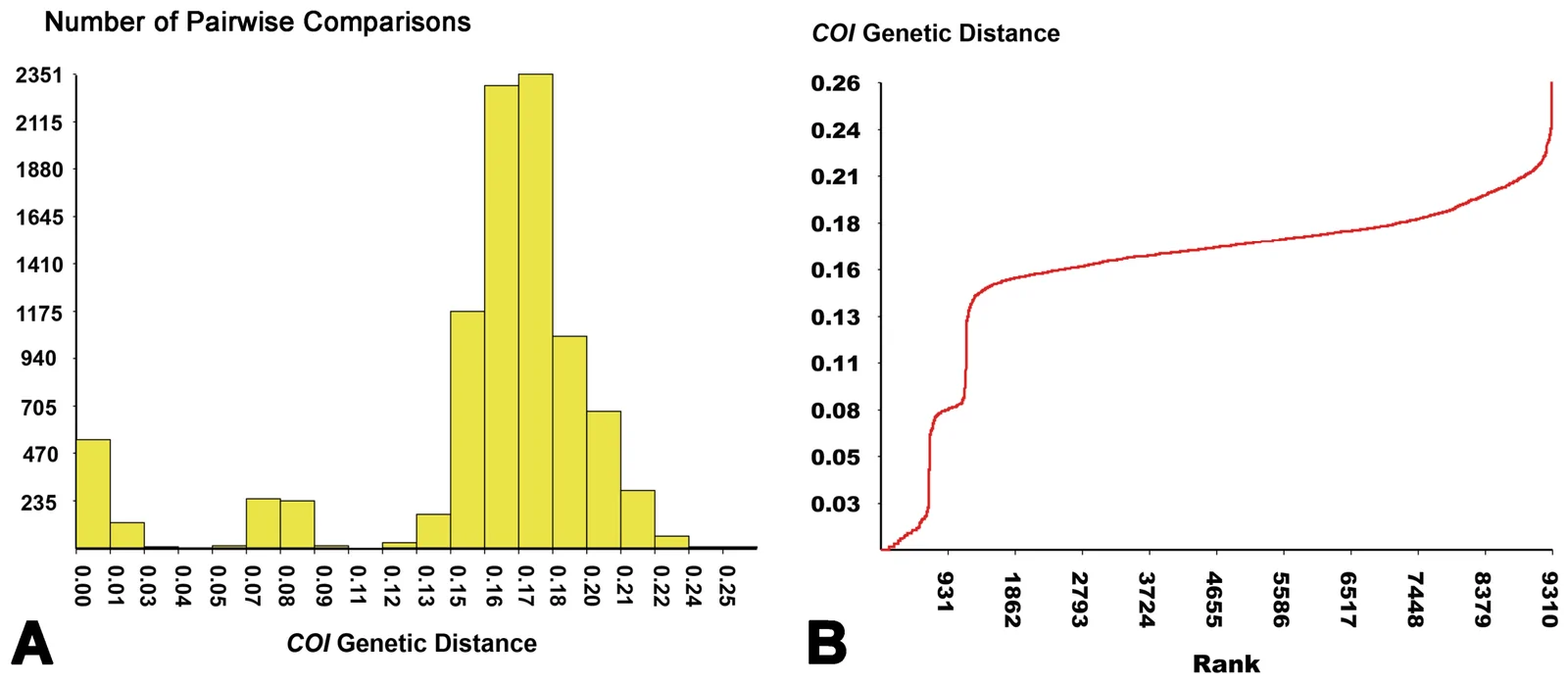
ABGD analysis of *COI* sequences for *Neozavrelia*. (**A**) Histogram of pairwise K2P genetic distance frequencies; (**B**) ranked genetic distance curve identifying the barcode gap threshold.

ABGD analysis of *COI* alignments revealed a distinct barcode gap separating intraspecific and interspecific genetic variation (Figure 2). Pairwise K2P distances displayed a bimodal frequency distribution: most comparisons clustered at low divergence values corresponding to within-lineage variation, while a clear discontinuity separated these from higher inter-lineage distances (Figure 2A). The ranked distance curve further validated the existence of a stable partitioning threshold for molecular species delimitation (Figure 2B).

ABGD delimited 26 molecular operational taxonomic units (MOTUs; Table S2). Pairwise K2P genetic distances across the full dataset ranged from 0.000 to 0.215. The maximum intraspecific divergence reached 0.118 between sequences XL1859 and LC329147 within Group 5, which were collected from geographically distant regions (Hainan, China and Toyama Prefecture, Japan, respectively), while the minimum interspecific distance was 0.115 (between Group 26 and Group 20) (Table S3). Although marginal overlap existed between maximal intraspecific and minimal interspecific distances, inter-MOTU divergence was consistently higher than within-MOTU variation overall.

### 3.3. Phylogenetic Clustering Validated by NJ Tree

**Figure 3:**
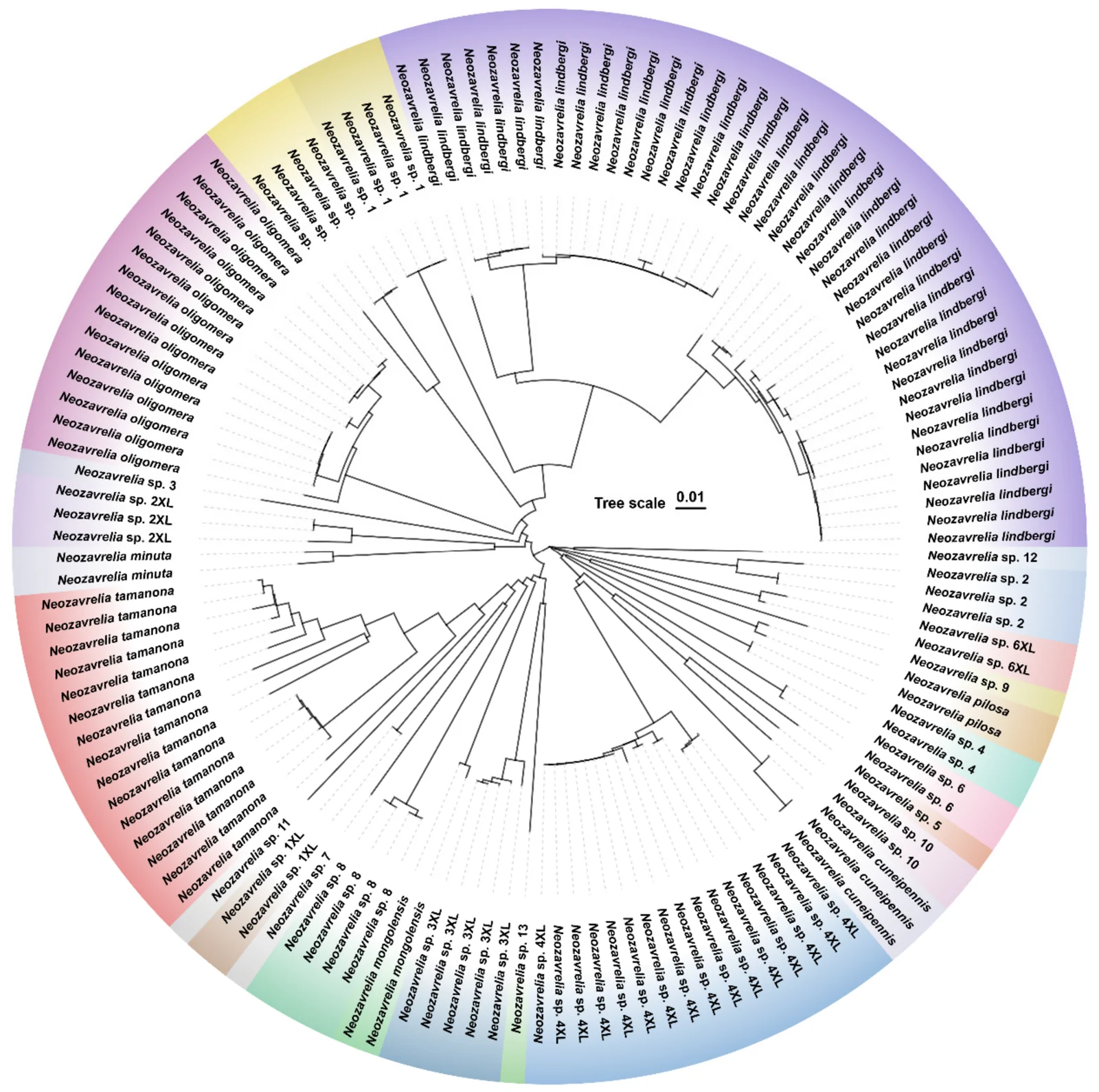
Neighbor-Joining tree constructed based on the *COI* DNA barcodes of *Neozavrelia*.

To visually verify the MOTU boundaries defined by ABGD, we reconstructed a neighbor-joining (NJ) tree based on *COI* K2P genetic distances (Figure 3). The tree topology exhibited strong congruence with the ABGD delimitation scheme. Every MOTU recovered by distance-based partitioning formed a well-supported monophyletic clade, with bootstrap support values exceeding 70% for nearly all lineages. The color-labeled circular tree clearly distinguishes the 26 independent genetic lineages and highlights deep genetic divergence between different MOTUs of *Neozavrelia*.

### 3.4. Environmental Variation and PCA Structure

**Figure 4:**
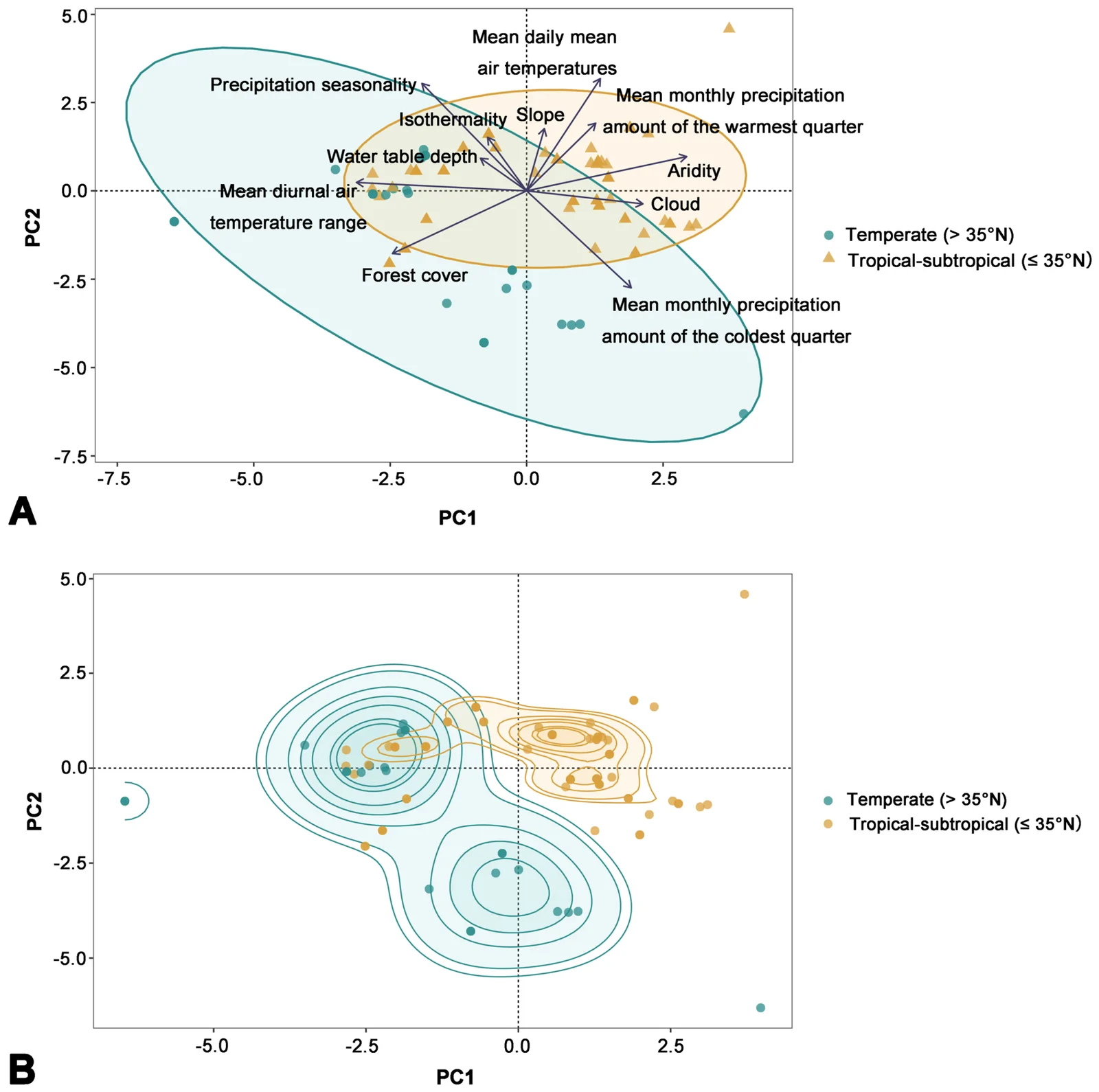
Ecological niche differentiation of *Neozavrelia* along standardized environmental gradients. (**A**) PCA biplot based on standardized environmental predictors. Symbols represent individual sampled individuals grouped by climatic zone: cyan dots = temperate assemblages (>35° N), orange triangles = tropical–subtropical assemblages (≤35° N). Colored ellipses represent the distribution ranges of the two climatic assemblages in PCA environmental space. Arrows denote loading vectors of 11 collinearity-filtered environmental variables. (**B**) KDE of temperate and tropical–subtropical assemblages in the PCA environmental space. Contour lines represent the density distribution of occurrences, with higher-density regions indicating greater concentration of samples. The KDE contours illustrate differences in ecological niche occupancy and the degree of niche overlap between the two climatic assemblages.

A total of 132 samples with complete geographic and environmental data were retained for environmental analyses after filtering missing values. Of these, 24 samples were from temperate regions (>35° N) and 108 from tropical–subtropical regions (≤35° N). After collinearity screening, 11 environmental variables were retained for downstream analyses (all VIF < 5). PCA showed that the first two principal components explained 48.7% of total environmental variation (PC1: 29.0%, PC2: 19.7%) (Figure 4A). PC1 was mainly associated with gradients in temperature seasonality, frost frequency, and humidity-related variables, while PC2 reflected variation in precipitation seasonality and cold-season temperature conditions.

The biplot vectors exhibited clear differentiation in variable loading strength and directional grouping. Long vectors with strong loadings dominated the PCA axes: precipitation seasonality and mean diurnal air temperature range drove negative PC1 variation, with forest cover showing moderate negative PC1 loadings; mean monthly precipitation amount of the coldest quarter defined negative PC2 while carrying a positive PC1 component; mean daily mean air temperatures, aridity, annual cloud cover, and mean monthly precipitation amount of the warmest quarter clustered along positive PC1. In the PCA ordination biplot, temperate and tropical–subtropical specimens exhibited partial ecological separation along the PC1 axis. Temperate localities (>35° N) predominantly occupied the negative PC1 dimension, while tropical–subtropical sites (≤35° N) aggregated on the positive PC1 side. Kernel density estimation (KDE) contours further visualized the clear niche segregation between the two climatic assemblages, with minimal overlap in two-dimensional environmental space (Figure 4B).

### 3.5. Genetic Differentiation and Environmental-Geographic Associations

**Figure 5:**
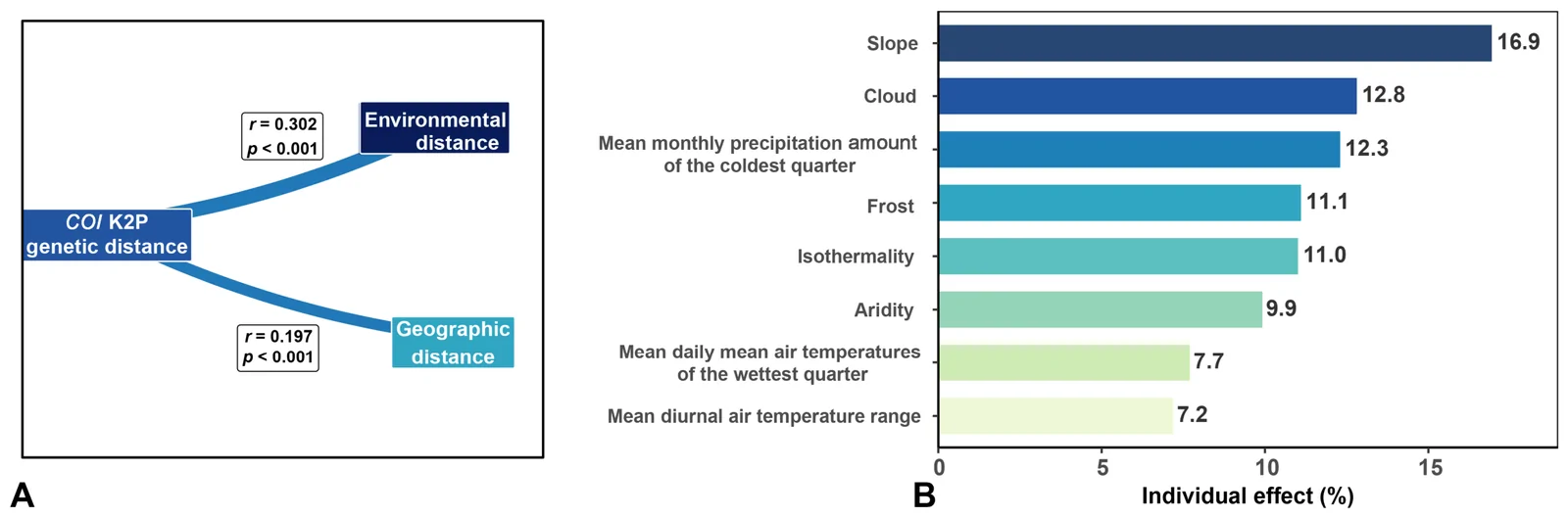
Relative contributions of environmental and geographical predictors to genetic differentiation of *Neozavrelia*. (**A**) Schematic diagram of partial Mantel test results showing correlation coefficients between *COI* K2P genetic distance and environmental/geographic distance after mutual control; (**B**) horizontal bar chart displaying the independent explanatory proportion (%) of each environmental variable via hierarchical partitioning analysis.

Simple Mantel tests detected significant positive correlations between *COI* K2P genetic distance and both environmental distance (*r* = 0.417, *p* < 0.001) and geographic distance (*r* = 0.356, *p* < 0.001). Partial Mantel tests further disentangled their independent effects (Figure 5A): after accounting for spatial isolation, environmental distance retained a strong significant correlation with genetic divergence (*r* = 0.302, *p* < 0.001); by contrast, the spatial correlation weakened substantially when environmental variation was statistically controlled (*r* = 0.197, *p* < 0.001).

Distance-based redundancy analysis (db-RDA) yielded a globally significant model (*F* = 3.489, *p* < 0.001), which explained 17.3% of total genetic variation across *Neozavrelia*. When geographic effects were partialled out, environmental predictors independently accounted for 15.8% of genetic structure (*F* = 3.295, *p* < 0.001). Variation partitioning revealed the relative unique and shared explanatory power of the two factor sets: environmental variables alone explained 34.4% of genetic differentiation, pure geographic distance explained only 3.8%, their joint interactive contribution reached 3.1%, and the remaining 58.7% of genetic variance could not be attributed to measured spatial or climatic predictors.

Hierarchical partitioning quantified the independent explanatory proportion of genetic variation attributable to each environmental variable (Figure 5B). Slope showed the largest independent explanatory share for intraspecific genetic structure (16.9%), followed by annual cloud cover (12.8%), mean monthly precipitation amount of the coldest quarter (12.3%), and frost frequency (11.1%). Isothermality, aridity, mean daily air temperatures of the wettest quarter, and mean diurnal air temperature range jointly contributed moderate to weak explanatory power.

## 4. Discussion

### 4.1. Genetic Diversity and Cryptic Lineages in Neozavrelia

ABGD analysis delineated 26 molecular operational taxonomic units (MOTUs) within *Neozavrelia*, with most MOTUs forming well-supported monophyletic clusters in the NJ tree. These results indicate that *COI* barcodes provide effective resolution for identifying major genetic lineages within the genus and reveal considerable genetic diversity requiring further taxonomic investigation.

Traditional taxonomy of *Neozavrelia* has relied primarily on morphological characters of adult males, particularly genital structures [18,21,23,24]. However, morphological approaches are often constrained by subtle interspecific variation, small body size, and difficulties in associating life stages, which may lead to underestimation of species diversity. In this context, *COI* barcoding provides complementary evidence for detecting genetically distinct lineages that are not readily distinguishable morphologically [63,64]. The clear genetic partitioning among multiple MOTUs in the present study indicates previously unrecognized genetic diversity within *Neozavrelia*, consistent with ongoing species discoveries and taxonomic revisions reported from East Asia, the Russian Far East, and other regions.

Although most MOTUs showed clear genetic separation, a small number of lineages exhibited partial overlap between intraspecific and interspecific distances, with the maximum intraspecific divergence (0.118) slightly exceeding the minimum interspecific divergence (0.115). Such patterns may reflect incomplete lineage sorting, recent divergence, or historical introgression, which are commonly reported in rapidly radiating insect groups [65,66]. However, *COI*-based species delimitation should be interpreted cautiously because mitochondrial markers may not always accurately represent species boundaries [67]. Discordance between mitochondrial and nuclear genomes caused by introgression, incomplete lineage sorting, or other evolutionary processes can result in misleading patterns of species relationships and diversity estimates [68]. Conversely, closely related species may exhibit limited mitochondrial divergence despite substantial differentiation in nuclear genomes, further highlighting the importance of integrative approaches for species delimitation [69]. Therefore, although *COI* barcodes effectively capture the general diversification framework of *Neozavrelia*, MOTUs with deep internal divergence or ambiguous barcode gaps require integrative validation combining morphological examination, nuclear genetic markers, ecological evidence, and broader geographic sampling [65].

In particular, Group 5 exhibited remarkably high genetic divergence, with the maximum distance occurring between specimens from Hainan, China (XL1859) and Toyama Prefecture, Japan (LC329147). This pattern suggests substantial geographic structuring within Group 5, potentially resulting from long-term isolation among geographically separated populations. However, Group 5 was retained as a single MOTU because all sequences formed a well-supported monophyletic cluster in the NJ tree and were consistently recognized as one partition by ABGD. Splitting this group solely based on *COI* divergence may overestimate lineage diversity, because deep mitochondrial differentiation can also arise from historical demographic processes, geographic isolation, or lineage-specific evolutionary patterns without necessarily indicating multiple independently evolving species. Nevertheless, the pronounced divergence within Group 5 highlights this lineage as a priority target for future integrative taxonomic studies incorporating morphological reassessment, nuclear markers, and expanded geographic sampling.

### 4.2. Environmental Heterogeneity and Genetic Differentiation

Mantel and partial Mantel tests revealed significant associations between genetic differentiation and both environmental and geographic distances, with environmental distance showing a relatively stronger correlation [70,71]. After controlling for geographic effects, the genetic–environment relationship remained significant, whereas the genetic-geographic association weakened when environmental variation was considered.

These results suggest that both spatial isolation and environmental heterogeneity contribute to genetic structuring in *Neozavrelia*, with environmental variation potentially playing a more prominent role. This pattern is consistent with the ecological characteristics of the genus, which is associated with freshwater microhabitats such as springs, streams, rivers, and hygropetric seepages [18,20,24,26].

Compared with terrestrial insects, aquatic insects are more constrained by hydrological connectivity, microhabitat stability, and water temperature fluctuations [63,72]. For small-bodied chironomids, limited dispersal ability combined with habitat discontinuity may correlate with greater genetic divergence across environmentally heterogeneous landscapes [73]. However, a substantial proportion of unexplained variation suggests that additional historical and ecological processes may also shape the observed genetic patterns.

db-RDA further supported the role of environmental factors. After accounting for spatial autocorrelation, environmental variables still significantly explained genetic variation, indicating an independent environmental effect on lineage differentiation. Variation partitioning showed that environmental factors explained a higher proportion of genetic variation than geographic factors, highlighting environmental heterogeneity as a strongly correlated factor linked to *Neozavrelia* genetic differentiation. Nevertheless, a considerable fraction of variation remained unexplained, suggesting potential influences from historical climatic fluctuations, river network topology, local water chemistry, vegetation cover, microhabitat types, and sampling bias [10,74].

### 4.3. Environmental Factors Correlated with Lineage Genetic Differentiation

Hierarchical partitioning analysis identified slope, annual mean cloud cover, mean monthly precipitation amount of the coldest quarter, and frost frequency as environmental variables showing the strongest independent associations with genetic differentiation in *Neozavrelia*. These results suggest that lineage genetic differentiation is significantly associated with variation in topography, cold-season hydrological conditions, humidity stability, and winter thermal regimes.

Slope showed the strongest independent association with genetic differentiation among the evaluated environmental variables, suggesting that topographic variation may be an important correlate of lineage structure in *Neozavrelia*. For *Neozavrelia* restricted to springs and hygropetric seepages, slope gradient directly controls flow velocity, substrate moisture retention, and surface runoff stability. Steep slopes generate fast, transient seepage habitats, while gentle slopes maintain persistent shallow water substrates suitable for larval feeding and development. Strong topographic filtering may correlate with reduced gene flow between sites with divergent slope conditions, which is associated with greater genetic differentiation across populations [75].

Annual mean cloud cover and mean monthly precipitation amount of the coldest quarter were also strongly associated with genetic differentiation. Cloud cover correlates with solar radiation and surface evaporation, sustaining stable humid microhabitats and reducing desiccation risk for moisture-dependent larvae. Cold-season precipitation sustains base flow of spring and seepage systems during cool periods, potentially reducing the probability of habitat drying in winter when surface runoff is limited [76]. Areas with sufficient cold-season precipitation tend to maintain year-round viable habitat connectivity, whereas regions with scarce cold-season precipitation show seasonal habitat fragmentation associated with greater genetic separation among lineages.

Frost frequency showed a strong association with genetic differentiation, suggesting that winter thermal conditions may contribute to differences in lineage distribution and genetic structure. Frequent frost events are correlated with reduced larval survival and narrower species distribution limited to frost-free or low-frost microrefugia; divergent frost regimes may correspond to different thermal environments that are associated with greater genetic separation. Other moderate-weak predictors including isothermality, aridity and wettest-quarter temperature further modulate local thermal and hydrological conditions, but their independent roles in shaping lineage divergence are secondary to topography, cold-season precipitation, cloud cover and frost exposure [77,78].

Overall, these results suggest that *Neozavrelia* lineage genetic differentiation is jointly correlated with topographic microhabitat structure and cold-season hydrological/winter thermal regimes, rather than warm-season precipitation or groundwater depth as previously hypothesized. Fine-scale topographic variation and cold-climate conditions represent environmental gradients associated with ecological differentiation and genetic structuring across the global distribution of this genus.

### 4.4. Environmental Differentiation Across Latitudinal Regions

PCA ordination revealed clear environmental differentiation between tropical–subtropical and temperate populations of *Neozavrelia*. Temperate populations clustered along the negative PC1 axis, associated with frequent frost events, high seasonality, and large diurnal temperature variation. In contrast, tropical–subtropical populations occupied the positive PC1 axis, characterized by warm, humid, low-frost, and high-precipitation conditions. Although partial overlap existed, two-dimensional KDE analysis indicated distinct niche density separation, suggesting divergent environmental preferences among populations from different latitudes.

These results suggest that *Neozavrelia* occupies a broad range of environmental conditions, with different lineages associated with contrasting climatic environments. Temperate lineages were associated with colder, more seasonal, and frost-prone environments, whereas tropical–subtropical lineages were associated with warmer, more humid, and hydrologically stable conditions [79]. This niche differentiation helps explain the wide geographic distribution of *Neozavrelia* across the Palaearctic, Oriental, and other regions, and indicates that the genus comprises multiple lineages with heterogeneous ecological preferences.

Consistent with previous records of *Neozavrelia* in hygropetric microhabitats, our results further highlight environmental differentiation along temperature and moisture gradients. Some lineages are associated with stable moist habitats, whereas others tolerate more seasonal and thermally variable environments [80]. Such niche differentiation likely promotes genetic isolation across environmental gradients and facilitates lineage diversification in *Neozavrelia*.

However, these environmental differentiation patterns should be interpreted with caution because the temperate group was represented by a substantially smaller number of samples than the tropical–subtropical group (24 versus 108 samples). In addition, many temperate records, particularly from Europe, were derived from single specimens available in BOLD, which may not fully represent the environmental and genetic diversity of Palaearctic populations. Therefore, the observed association between frost frequency and temperate lineages should be considered preliminary and requires validation with broader geographic sampling and increased population-level representation.

## 5. Conclusions

Based on broad-range global sampling and publicly available BOLD database sequences, this study constructed a comprehensive *COI* barcode reference dataset for *Neozavrelia*. By integrating ABGD molecular species delimitation, NJ phylogenetic inference, and multi-dimensional environmental association analyses, we systematically evaluated the genus’s genetic diversity, lineage diversification patterns, and underlying ecological drivers. Our results revealed high *COI*-based genetic diversity within *Neozavrelia*, with 26 MOTUs representing distinct genetic clusters supported by phylogenetic evidence. These results indicate that *COI* barcodes are effective for revealing major genetic lineages within *Neozavrelia* and suggest the presence of previously unrecognized cryptic diversity that requires further validation through integrative taxonomic approaches.

Multi-scale environmental analyses confirmed that genetic differentiation in *Neozavrelia* shows significant correlations with both geographical isolation and environmental heterogeneity, with environmental predictors explaining a far larger proportion of observed genetic variation than pure spatial distance. Hierarchical partitioning identified slope, annual cloud cover, mean monthly precipitation amount of the coldest quarter, and frost frequency as environmental variables showing the strongest independent associations with lineage genetic differentiation. These variables were significantly associated with genetic differentiation and may reflect differences in topographic conditions, hydrological stability, atmospheric humidity, and winter thermal regimes. PCA-based ecological niche analyses further revealed significant environmental niche segregation between tropical–subtropical and temperate populations, reflecting differences in environmental associations among lineages across broad latitudinal gradients.

Overall, this study illustrates that lineage genetic differentiation of *Neozavrelia* is jointly correlated with environmental filtering and weak geographical isolation, with topography, cold-season precipitation, cloud cover, and frost regimes representing important environmental variables associated with genetic divergence. Our findings substantially expand the global molecular baseline dataset for *Neozavrelia* and provide novel insights into genetic differentiation patterns, ecological associations, and biogeographic diversification processes of chironomids specialized to small freshwater seepage microhabitats. The *COI* barcode library generated here provides fundamental resources for future taxonomic revisions, cryptic species discovery, regional freshwater biodiversity surveys, and long-term ecological monitoring targeting *Neozavrelia*. Future research incorporating expanded geographic sampling, multi-locus or genome-scale molecular data, and fine-scale in situ microhabitat hydrological measurements will help further disentangle the relationships between environmental adaptation and evolutionary diversification in this genus.

## Figures and Tables

**Table 1:** PCR Reaction Mixture.

Protocol	Reagent	Volume
PCR Reaction Mixture	2× Es Taq Master Mix (Dye)	12.5 µL
	LCO Primer (10 μmol·L^−1^)	0.5 µL
	HCO Primer (10 μmol·L^−1^)	0.5 µL
	ddH_2_O	9.5 µL
	DNA	2 µL

**Table 2:** PCR Amplification Program.

Step	Temperature	Purpose	Time	Cycle(s)
Pre-denaturation	94 °C	Initial Denaturation	2 min	1
First amplification stage				5
	94 °C	Denaturation	1 min	
	49 °C	Annealing	30 s	
	72 °C	Extension	50 s	
Second amplification stage				35
	94 °C	Denaturation	1 min	
	51 °C	Annealing	90 s	
	72 °C	Extension	1 min	
Final Extension	72 °C	Final Extension	5 min	1
Hold	4 °C	Storage	Forever	1

## Data Availability

The data presented in this study are available in the Barcode of Life Data System (http://www.boldsystems.org, accessed on 19 May 2026) under the dataset “*COI* barcodes of *Neozavrelia* until 2026 (DS-26NEOZ)”.

## Supplementary Materials

The following supporting information can be downloaded at: https://www.mdpi.com/article/10.3390/insects17080814/s1, Table S1. Sampling metadata for all *Neozavrelia* specimens sequenced in this study. Table S2. Molecular operational taxonomic units (MOTUs) of *Neozavrelia* delimited by ABGD analysis, with corresponding taxonomic assignments. Table S3. Genetic distance matrix.
